# Implementation of NAO Robot Maze Navigation Based on Computer Vision and Collaborative Learning

**DOI:** 10.3389/frobt.2022.834021

**Published:** 2022-04-04

**Authors:** Daniela Magallán-Ramírez, Jorge David Martínez-Aguilar, Areli Rodríguez-Tirado, David Balderas, Edgar Omar López-Caudana, Carlos Francisco Moreno-García

**Affiliations:** ^1^ Tecnologico de Monterrey, School of Engineering and Sciences, Monterrey, United Kingdom; ^2^ Robert Gordon University, School of Computing, Aberdeen, United Kingdom

**Keywords:** robot navigation, computer vision, mapping, NAO robot, educational innovation

## Abstract

Maze navigation using one or more robots has become a recurring challenge in scientific literature and real life practice, with fleets having to find faster and better ways to navigate environments such as a travel hub, airports, or for evacuation of disaster zones. Many methodologies have been explored to solve this issue, including the implementation of a variety of sensors and other signal receiving systems. Most interestingly, camera-based techniques have become more popular in this kind of scenarios, given their robustness and scalability. In this paper, we implement an end-to-end strategy to address this scenario, allowing a robot to solve a maze in an autonomous way, by using computer vision and path planning. In addition, this robot shares the generated knowledge to another by means of communication protocols, having to adapt its mechanical characteristics to be capable of solving the same challenge. The paper presents experimental validation of the four components of this solution, namely camera calibration, maze mapping, path planning and robot communication. Finally, we showcase some initial experimentation in a pair of robots with different mechanical characteristics. Further implementations of this work include communicating the robots for other tasks, such as teaching assistance, remote classes, and other innovations in higher education.

## 1 Introduction

An autonomous system for maze navigation must be able to take real-time decisions for different and sometimes unexpected problems that may occur. For instance, a robot might be aware of its environment by using different sensors, data processing algorithms or other methods. In spite of this, one of the most widely used methodologies consists of collecting the information that the robot needs by means of the embedded cameras. This kind of techniques have been implemented not just for high school/university competitions, as shown in Horn (1986), but also in practical applications, such as airport navigation by a fleet of robots, as presented in Cortés et al. (2016). The problem of using other sensors (e.g. sonar or infrared lights) is that these require conditional programming, which particularizes each solution to a specific type and version of robot. Therefore, academia and industry work hard to find methods that can be applied to different robotic platforms and that would allow real-life applications in areas such as education, industry 4.0, health, among others.

This paper presents the test and comparison of different methods that will conform an autonomous maze solution system for a pair of NAO^1^ robots. This proposal will enable them to navigate a maze in an autonomous way by means of four main parts: 1) camera calibration, which allows the acquired images from the first robot to be preprocessed and cleaned prior to their use; 2) mapping, which consists in vision algorithms to analyze the images and generate an internal map; 3) path planning, which enables the first robot to make decisions about the navigation to get out in the most optimal way and; 4) communication, which transmits the generated knowledge to allow a second robot to solve the considering its mechanical characteristics. We have selected this particular robot brand since it can be programmed using a multi-platform environment called *Choregraphe*, which integrates script from languages such as C++, YAML and *Python*. Moreover, NAO robots possess a variety of sensors which can be integrated to our solution in future work, such as touch sensors, omni-directional microphones and ultrasonic sensors^2^. We developed this work in the 6^*th*^ generation of these robots, which are widely used in education and research.

This is an extension on the paper presented in Rodriguez-Tirado et al. (2020), where we introduced an initial proposal which would allow the navigation of robot into a maze by means of the aforementioned pipeline framework. Nonetheless, in this paper we present the following contributions:

1. We thoroughly review the state of the art in NAO robot navigation.
2. We present an updated and easy to follow pipeline methodology for NAO robot maze navigation.
3. We justify the reasons to choose the selected methods and validate each step.

The modifications implemented in each step have as final objective to fully integrate the system. Finally, this paper presents the migration of this final integration into a real environment and its applicability in higher education.

After the introduction in Section 1, this paper is organized as follows. Section 2 contains a literature review of the existing investigations and applications of similar methodologies in various scenarios. Section 3 describes how to interconnect the four modules proposed to solve robot navigation and introduces the methods used in each module. Section 4 presents bot the results of all tests in each step, and the integration of such modules. Afterwards, Section 5 analyses these results. Finally, Section 6 is reserved for conclusions and future directions.

## 2 Related Work

### 2.1 Related Work on Camera Calibration

To improve the response time of a fleet of NAO robots playing football soccer, Kastne et al. (2014) implemented a kinematic calibration within the robots. Authors used a calibration method originally proposed in Zhang (2000), which will also be considered in our work. This method is based on a chess pattern, and the calibration result was helpful to compensate the errors of the NAO robots legs, which allowed these robots to be able to better kick the ball. Another objective of such work was to perform a fast calibration before a match, thus resulting on an elapsed time of about 590 s. According to that paper, in a 10 minute match (without the calibration) the robots fell an estimate of eleven times per match, and after the calibration, this was reduced to six times.

NAO robots use camera calibration for other applications, such as portrait sketching as shown in Singh and Nandi (2016). In this implementation, it was necessary to change the plane from 3D to 2D by transforming the effector position into the NAO, so that the robot, instead of having to compute the coordinates of a tri-dimensional plane, could just perceive the *x* − and *y* − coordinates to sketch a portrait. Authors evaluated three solutions for the 3D-2D transformation, which were: 1) fundamental matrix; 2) 4-point calibration and an; 3) an artificial neural network (ANN) based regression analysis. Comparing the experimental results of the three solutions, the third option was the best since it derived on less square error. Conversely, the method with the lowest computational time was the 4-point calibration.

### 2.2 Related Work on Path Planning

One of the most widely used solutions used in this domain the *Tremaux* algorithm, which has been subsequently applied in literature by different authors. For instance, Li and Annaz (2014) implemented this approach with the purpose of having a mobile robot in a virtual environment be able to navigate and find the minimum cost in the shortest time. As a result, the robot was able to indicate the shortest path, without actually being able to navigate it or to communicate the solution to a second robot. An important feature of this work is that the robot was given the location of the exit; something that may not necessarily be our case.

Kumar et al. (2018) used path planning and other algorithms aimed at avoiding obstacles to solve maze routes, by means of a linear regression method. The robot was trained with 500 different scenarios. In a second part of the work, a second robot with the same algorithm was introduced to the same route. The main purpose was that both robots could solve the puzzle simultaneously without any collisions. The obstacles consisted of cylinders, and the robots had to follow an establish path. Therefore, robots just had to get from one point to another with a given trajectory that would be adjusted depending of physical obstructions. Although this holds similarity to our proposal, authors do not specify whether the communicated knowledge to the second robot considered different mechanical properties of that robot.

Wikström and Sjögren (2016) worked on how a robot can process its environment to map and navigate through a maze. Using an infrared light sensor, the robot was able to obtain information from its surroundings. *Python 2* was used to program the mapping and path planning modules. In this thesis, the efficiency between the Wall Followers and the *Tremaux* algorithm were compared. It was concluded that the latter was more effective to solve the task at hand. This work has some similarities with our proposal, nonetheless no computer vision algorithms were implemented in that case, which effectively showed a reduction in reliability and accuracy of the obtained solution.

More recently, Kashyap et al. (2020) presented a hybrid technique for path planning in static and dynamic terrains using NAO humanoid robots. Authors proposed the hybridization of two techniques: Dynamic Window Approach (DWA) and Teaching-Learning Based Optimization (TLBO). This method requires the location of the obstacles as the input target, so that DWA can decide the optimal velocity for the robot to traverse in order to dodge the obstacles. Afterwards, TBLO operates using a teacher-learner methodology (within the same NAO robot) to teach itself how to effectively navigate the terrain. This method showed promising results in terms of navigating through rugged terrain, although the requirement of the obstacles as the input make it less attractive for undiscovered maze navigation solving.

The most recent work presented in this domain was published last year by Muni et al. (2021). Authors suggested Prim’s algorithm, which is a Minimum Spanning Tree (MST) greedy method which generates sets from a weighted graph, picking the least weighted edge to create non incidences and thus, a path to be traversed. These edges are then transmitted to the NAO robot, showing a decrease of 6% in deviations of navigational parameters. This method appears to favor path length cover against path time taken, which is the opposite from what it is required in maze navigation contests or emergency evacuation situations.

### 2.3 Related Work on Communication Protocols

Simoens et al. (2018) defined and explained the potential benefits of the Internet of Robotic Things (IoRT). Some of the key benefits of this area of knowledge are perception, motion, manipulation, interaction, adaptability, configuration and decision autonomy. In particular, the latter has the ability of determine the best action for a fleet of robots to undertake. In our work, we propose to connect the NAO robots by means of commercial platforms such as *Ubidots*, in which the data recall, such as the instruction for the robot, can be download and transfer easily. This in effect has some relation to the IoRT concept.

Bechon and Slotine (2013) implemented a swarm code to coordinate the actions of eight NAO robots. As a result of this work, the fleet was able to communicate among them by using a global variable to synchronize the whole NAO robot group in a dance choreography. The robots were synchronized using the Network Time Protocol (NTP), getting all NAO robots synchronized. Distance between the robots was an important factor; while it increases, also the time to detect and correct the next position does. Because of that, it was necessary to implement a mesh network where all robots had access to each other, so that each robot was able to communicate with their closest neighbor. This type of architectures are interesting for a future implementation of this work, provided that we need to synchronize multiple robots simultaneously.

### 2.4 Other Systems

Evidently, maze navigation can be applied to multiple other robotic standards in the industry. For instance, Gul et al. (2019) explained how different navigation techniques can be applied into a mobile robot to follow a dynamic path. The robot had to go from the start to the end by avoiding obstacles between the points and analyzing a 2D plane. Some of the algorithms used were the Dijkstra method (which will be explored in this paper). This was found to be a simple, effective and less time and computational complex method with a very high efficiency for the task.

Delfin et al. (2016) aimed at localizing and helping a NAO robot to navigate into an environment by using a visual memory which consisted on a set of key images. First, the NAO got into the environment and tried to solve by mimicking a human. In parallel, images were acquired and used to generate a graph in which a path planning method was applied to help the robot optimize it trajectory in the environment. To our knowledge this is the closest work in literature to our proposal. Nonetheless, we have the added complexity of a second robot which has to solve the same challenge based on a shared knowledge and considering different mechanical characteristics.

## 3 Methodology

The system starts by establishing the communication between the robot and the computer which controls the robot. Then, camera calibration is applied before the robot gets into the maze, as the robot must use the cameras to solve it. Afterwards, the robot is ready to start the maze, and thus, the NAO robot will collect and process an image to begin generating a section of the maze map. That section of the map will then be used in path planning related tasks to tell the robot what step to take in order to exit the maze. When the robot finishes its movement, it will continue acquiring these images to create the internal map and advance according to the process that was mentioned before. This process will repeat until the image that the robot detected depicts the exit. When the robot exits, it will then know a route to exit the maze, and will also have acquired an internal map of the part of the maze where it passed. This information will be used to select the best path according to the robot’s experience, and transmit this to the second one. Due to the nature of this process, we will present the four main parts sequentially.

### 3.1 Camera Calibration Methodology

Camera calibration is a process that helps minimize image imperfections caused by of intrinsic and extrinsic camera parameters. This pre-processing step is used to accurately establish a relationship between 3D point (in the real world) and its projection in a 2D plane (image pixel). Moreover, the camera must be calibrated so that it captures images that are not distorted, and that the results obtained from the computer vision algorithms are less prone to errors. Furthermore, the robot will collect information such as the distances between the images, and thus the importance of this stage.

To achieve this, we implemented the method presented in Zhang (2000) which generates a calibration matrix to correct these distortions by taking multiple images of a well-defined object, in this case, a checkerboard. Some parameters such as brightness, saturation, and focal length must be manually adjusted to get the best possible image. Then, we generate the matrix by taking the corner patterns of the checkerboard which has dimensions of 6 × 5. Afterwards, we detect the corner and get the camera matrix and its distortion coefficients using modules contained in the OpenCV framework^3^. Finally, with the obtained information we generate a new matrix that will be used to un-distort the images.

### 3.2 Mapping Methodology

Robotic mapping refers to the ability of an autonomous robot to construct a map while being able to position itself within it. Navigation and mapping are closely related, and these concepts rely on different algorithms. However, while investigating the existing approaches, we found that most of them use different sensors (e.g. infrared sensors, odometers, GPS, etc.) which contrasts from our proposed scenario and therefore, adapting existing solutions is not an affordable task. As a result, we will be presenting a proprietary solution in this paper based on computer vision as the input.

Moreover, due to the COVID-19 worldwide pandemic, access to the NAO robots and the material for building up a test environment was restricted at the beginning of this project. From that perspective, the best solution was to take advantage of existing software tools to start the mapping module development. To this end, we used Coppelia Sim software^4^, which is a 3D model of a maze which was deployed virtually to record a video simulating the first robot traversing the maze. Then, that video was used to try out the map reconstruction with the proprietary code. To simulate the navigation a robot model that comes by default in Coppelia (called Ackerman), steering car having ease of control with the keyboard arrows was used. Additionally, a camera was attached to an Ackerman robot, and using OBS Studio video editing software, the video was recorded.

To achieve the mapping, the first step consists on applying the Canny algorithm presented in Canny (1986) to divide the image into different areas which allow to better control what the robot detects and maps.

The Canny algorithm needs many stages, with each one of them using a different operator to detect the edges of every image. In the first stage, the algorithm assumes that the image is affected by white noise, so it applies a first order Gaussian blur in the image to reduce the noise and to take out unwanted details and textures. The mathematical representation of the filter is based on two one-dimensional convolutions, one for horizontal axis and another for the vertical axis, merges together in Equation (1) as:

$$G_{\sigma }\left(x,y\right)=\frac{1}{2\pi \sigma^{2}}exp\left(-\frac{\left(x^{2}+y^{2}\right)}{2\sigma^{2}}\right)$$ (1)

Then, the Gaussian function is applied to the acquired image, as shown in :

$$g\left(x,y\right)=G_{\sigma }\left(x,y\right)\cdot I\left(x,y\right)$$ (2)

As shown in the last equation, the filter is a convolution between a two-dimensional mask or matrix and the target image *f* (*m*, *n*). Usually, the masks dimensions are about 5 × 5 and the result depends on the value assigned to *σ*. This value is chosen depending on a wanted behavior; for instance, a large value implies the detection of big scale edges, while a smaller value would lead to the detection of finer features.

**FIGURE 1:**
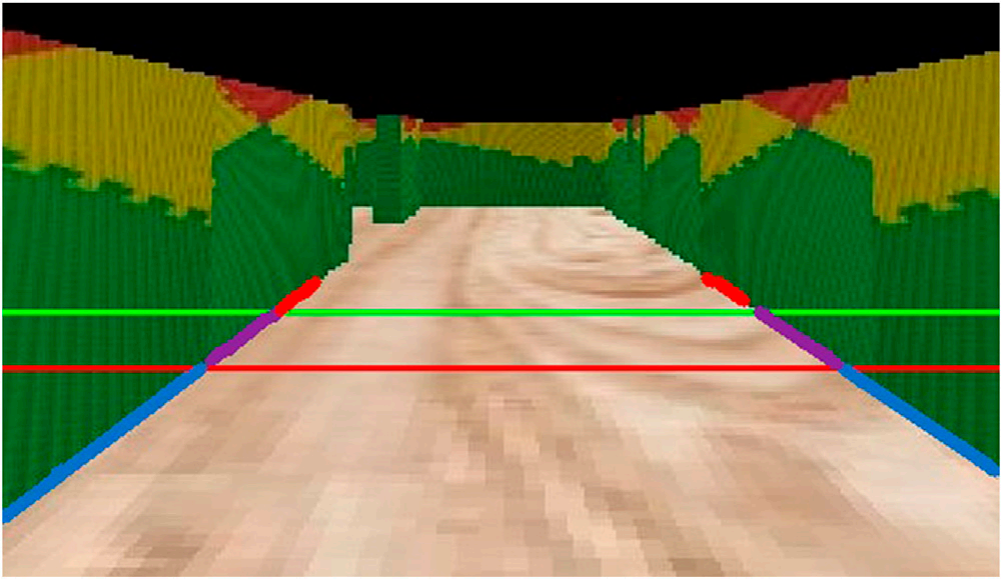
The three areas obtained by the robot, divided by green and red lines.

Figure 1 shows the three zones are obtained: 1) the top zone (i.e. the farthest); 2) the middle zone and; 3) the bottom zone (i.e. the closest). The robot uses just the middle zone to sketch the maze, and anything above or beyond zones one and three is discarded. To make the distinction between zones, the Canny edges of each section are stored in different arrays. This way, we can manage them individually as appropriate. Afterwards, the probabilistic Hough transform as presented in Hough (1962), Matas et al. (1998), followed by a merge algorithm^5^, is applied to each zone to find the lines which define the maze. This merging algorithm was considered since it makes the resulting array of probabilistic Hough lines smaller, thus making then easier to manipulate it frame by frame.

At this point, wall detection is carried out by changing the state of the two flags within the code, depending on whether the left or right wall is detected. If there is no wall, the Hough algorithm returns an empty array for the analyzed region, causing the flag to be deactivated within the code. Otherwise, if the array has elements, the flag is activated (provided that there is a wall). Then, the obtained middle lines (if any) are superimposed over a black image using a transform algorithm ^6^ which allows a better perspective of the space in front the robot, allowing it to determine approximate distances from the surrounding environment. For the estimation of distances, we used the Euclidean distances based on the pixels.

Subsequently in a new black image, the map sketching takes place by using a previously defined starting point, and then taking the distances to a 10 : 1 ratio, so that the entire map fits into a single image. The lateral lines that the robot visualizes in each frame are drawn from the starting point and when the sketching for this frame ends, the starting point is updated, taking as the new starting point the last coordinate of the drawn lines. In this way, the next segment of the map will be sketched just after what has already been plotted.

As the tests were conducted, it was decided that during the orientation changes, the mapping would stop drawing the maximum possible number of useless lines as possible; the rationale behind this will be explained in more detail in Section 4.2. Moreover, as the tests were made by means of a video, and there was no way of knowing when the robot was positioned, we had to define in advance when to stop mapping. This sketching process resumes once the presence of both walls is detected, as this indicates that it is rejoined into a corridor.

### 3.3 Path Planning Methodology

For this stage, we require a path planning method which allows the robot to navigate the maze without having a notion of where the exit is, while simultaneously intending to calculate the best possible route. Moreover, we need to deploy an algorithm which is simple enough to run in any operating system, any *Python* version (2 or 3) and that outputs a stream of simple data (i.e. binary directions) that can be transmitted from the central system to the master robot, and from the master robot to the slave robot, with low overhead and latency. Although some more recent and complex options have been implemented in NAO robots Kashyap et al. (2020) or Muni et al. (2021), due to the reasons stated in Section 2 we opted to use the Dijkstra Shortest Path algorithm presented in Ably et al. (2016). This method generates a tree of shortest paths from the starting point to all other points in the plane. Each possible step is a node that consists of two values, the distance to the start point and the last neighbor to get to that point with the least cost. There is a queue while it is not empty, as there are unknown possible nodes to visit. Also, there is a list that contains all the visited nodes with its characteristics. This method is based on discovering the new areas by extending a circular trajectory, and it is necessary to go back to the beginning to calculate distances. In this way, it ensures that the entity traversing the route will discover each possible step in all directions until the exit is found. Moreover, the second option considered was the Trémaux algorithm, which is widely known as an efficient method to get out of a maze, as shown in work such as Wilkins and Nelson (2008). It mark all the steps and the direction were the robot comes from. In this method, the navigator avoids traversing the same path twice by means of two lists. The first list contains all visited nodes in the order that they were found. The second list stores the path towards the exit. In principle, this second list will be a copy of the first one, but each time that the robot comes back to a node, all preceding nodes will be erased. For the actual implementation in the robot, we set priorities to four possible moves in the following order: 1) front step; 2) left step; 3) right step and; 4) back step.

**FIGURE 2:**
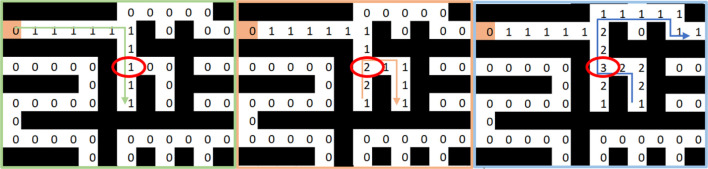
An image representing why a robot could pass 3 times in the same node while looking for an exit.

We made an exception to the Tremaux’s algorithm rule of never having to go back to a node twice because this could get the robot caught in some situations like the one shown in Figure 2. In that case, the node marked in red has been crossed more than once, however we allow the robot to cross again to find the exit, even when there is a priority to go to the nodes with less visits.

### 3.4 Communication Methodology

The NAO robot can communicate with a computer via Ethernet and WiFi to be programmed. In our case, WiFi was selected because the NAO robot will be in constant motion as it traverses the maze. Evidently, it would not be comfortable walking behind the NAO robot with the Ethernet cable connected to a computer. The connection via WiFi has the advantage of connecting with other web services, and any data can be transmitted and received directly between the computer and the NAO robot, such as temperature measure of some motor. This communication architecture is similar to Ad-hoc, but in this case, there is an access point that works as an intermediary. As mentioned before, Choregraphe is the official software developed by Aldebaran Robotics for the programming of the NAO robot, which uses the NAOqi framework so that it can be programmed in different programming languages, such as C ++, *Python*, Java, Matlab, Urbi and.Net, among others. To establish the wireless communication, the robot uses the registered port 9559. Note that registered ports are only used by some specific application or service^7^. The main technologies used for this stage, namely Ubidots, firebase and Google Drive API, are described in the following sections.

#### 3.4.1 Ubidots

Ubidots is a web page where data can be loaded through a JSON format file, then stored and viewed through many different widgets on a dashboard. This gives users an attractive user interface with a more professional touch without the user having to do any programming. This platform was used because it already has libraries created compatible with *Python* 2.7, facilitating communication with this web page. In addition, with a free account it allowed us to view up to ten simultaneous widgets on the dashboard. In addition, it offers you an authentication service through two credentials to be able to access your devices or registered projects. This platform was chosen because the first step to work communication between two NAO robots is that they send and receive a single value, for instance a Boolean, string or an integer.

#### 3.4.2 Firebase

Firebase is a real-time storage base, which does not require users to have deep knowledge about the management of servers or APIs. The libraries available are created for different programming languages, including *Python* 2.7, making handling and administration very simple. It is a BaaS service created by Google Cloud Platform, and therefore, it is compatible with any Google application. By creating a free account, users can store up to 1 GB of data, make 20,000 write requests and 50,000 read requests per day through JSON format files. This platform was used since the shared knowledge between NAO robots is a hexadecimal image or arrangement that represents the maze map and thus, the storage provided is sufficient for this transmission.

#### 3.4.3 Google Drive API

Google APIs allows the integration of an enormous variety of Google services, including Google Drive API. This is quite convenient, since from any application one can have the same control of a storage drive as when logged in from a browser. The programming languages supported are Java, Node.js and any version of *Python*. Google developed libraries for all of its services, facilitating integration with any application, and maintaining support for older *Python* versions, such as the one used by the NAO robot. This API was selected because Google Drive can store any type of file, which gives us greater freedom to choose the format of the maze map. For instance, if a hexadecimal string that represents an image is requested, a NAO robot can create and upload a CSV file, which can later be downloaded by the second NAO robot directly store the maze map image. Also, Google Drive allows 50,000 write requests per day, which is sufficient for our testing needs.

## 4 Test Bench

### 4.1 Camera Calibration Test Bench

**FIGURE 3:**
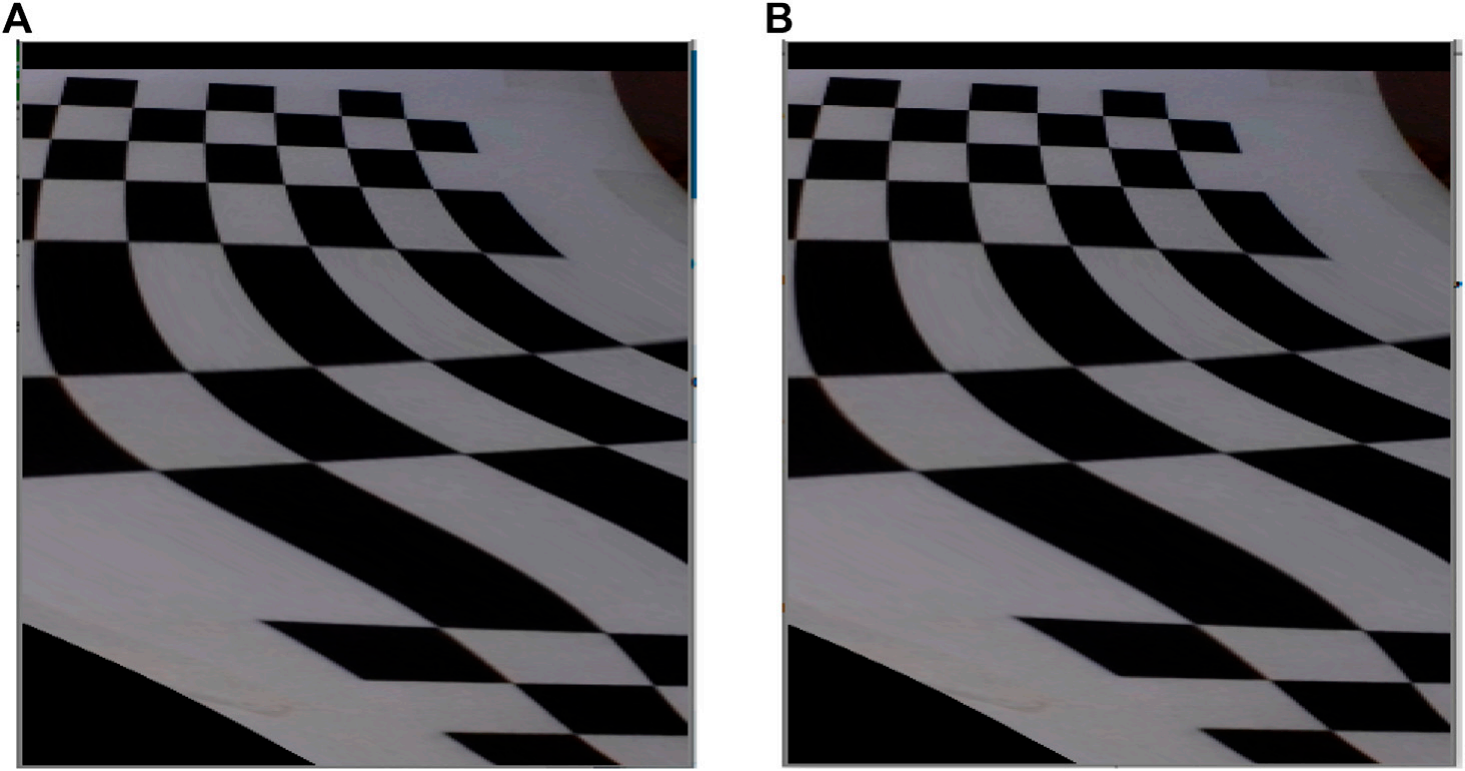
Calibration examples. **(A)** Un-distorted image with 10 samples. **(B)** Un-distorted image with 20 samples.

By taking several pictures of a checkerboard in different angles and under different lighting conditions, we generate an image database for the calibration. This calibration focuses on a ROI, which is the checkerboard itself. Evidently, the more images that are obtained, the better results that can be achieved. Nonetheless, we decided to stop at 51 images, since the matrix calibration parameter adjusted well to the image ones, as shown in Table 1. The dimension of the images are 680, ×, 420 and the calibration matrix use the center of the image, i.e. *cx* and *cy*, so base on those we calculate the error (%cx and %cy) between the real centers and the ones of the matrix. We can see that the error of both centers at 51 images is of less than 0.05. Moreover, Figure 3A,B show how the calibration works with different number of samples.

**TABLE 1:** Comparative table between the centers of the real image and those of the matrix (the best results achieved are highlighted in red).

Images	cx	cy	%cx	%cy
10	170.003	42.4261	0.469	0.823
20	159.479	28.9455	0.502	0.879
30	248.704	222.34	0.223	0.074
35	349.239	222.55	0.091	0.073
40	377.333	205.08	0.179	0.146
45	331	233	0.033	0.028
47	394	130	0.232	0.459
49	357	198	0.117	0.177
51	320.229	236.477	0.001	0.015

### 4.2 Mapping Test Bench

The Canny edge detection function contained in *Python*’s *skimage* package^8^ requires the configuration of three main parameter: two thresholds (one upper and one lower) and the aperture size for the Sobel operator. Therefore, the performance of this function was tested with different values to find the ideal configuration for this application. The first parameter that was experimented was the size of the Sobel operator, since this is used on the second step of the Canny algorithm after smoothing the image and so, it is the most significant parameter. This operator allows to acquire the orientation that each pixel is pointing at, by returning the value of the first derivative for the horizontal and vertical direction. We initialized the parameter with a value of 3, given that during our literature research we found that this is the standard size used; however, tests were run with aperture sizes of 5 and 7 as well. Ultimately, we confirmed that an aperture of three would yield the best performance.

To determine the upper threshold, we decided to start the tests with a threshold equal to 200, which was arbitrarily assigned. We noticed that its performance was not poor; however, the image returned showed many lines on both the walls and the floor. The threshold value was increased in steps of 10 until the optimal value was achieved. At a value of 250 the number of lines detected diminished. Furthermore, when reaching a threshold of 300 we observed that the images of the walls were significantly cleaner, and only one line was detected on the floor. From that threshold onward, the changes began to be less significant; when the threshold reached 400 we observed that a few lines were cleaned on the walls, but there was no significant changes. Finally, at a threshold of 500 no improvement was noticed, and thus this value (and any further) were discarded.

Therefore, it can be concluded that an acceptable value for the upper threshold could be found at the range of 250–400. It was then decided to use a value of 300 for this threshold, leaving it at an arbitrary but at the same time optimal value. On the other hand, in order to clean the image more and achieve better results, the lower threshold was adjusted as well. Experiments showed that the best image is obtained with a value of 150 since, if we using a filter of 200, certain empty spaces in the image begin to appear as full. This can cause problems in the lines detection, hence it was decided that it was better to leave the threshold at a 150; however, it is observed that the image is not completely cleaned.

In spite of the simulation results, the second filter was not discarded as an option for real time tests since, on a real environment, the floor actually presents many lines that could affect the performance of the algorithm.

**FIGURE 4:**
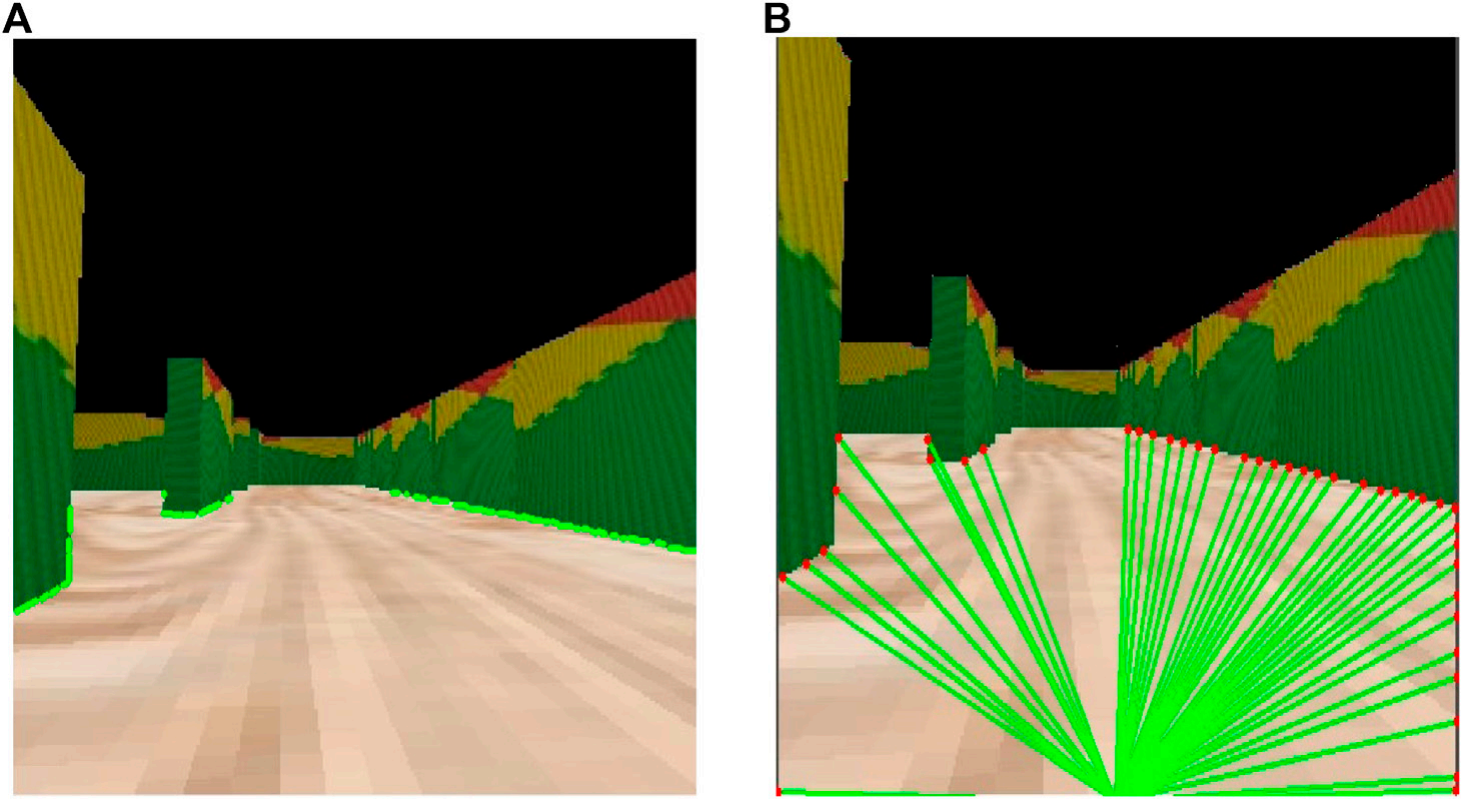
Comparison of line and corner algorithms. **(A)** Line detection algorithm performance. **(B)** Corner detection algorithm performance.

On the testing phase, the possibility of using a corner detection algorithm instead of a line detection algorithm was considered; however, corner detection had a reduced performance on detecting corners of the rendered image, and thus, in a real situation with smoother images, those characteristics would not be present. A comparative of both algorithms implemented in the simulation environment is shown in Figure 4A and Figure 4B respectively, and it can be seen that line detection seems to be more precise. Also, we did not want to limit the functionality of the system to a maze with corners, as we expect that this project can be implemented in different tasks and scenarios. For those reasons, this option was discarded and wee worked with lines detection using probabilistic Hough transform, as mentioned in Section 3.2.

For the sketching part, many tests were carried out and therefore, as considered this to be one of the most challenging stages. Firstly, as mentioned in Section 3.2 we had to stop mapping during turns because the sequence of the hallways was lost at these points.

Finally, it was found that, when a wall was not being detected, the distance was computed from the center to the first pixel of the image, which caused again useless line traces. This gave us yet another reason to keep using the flags which indicates the presence or absence of walls.

### 4.3 Path Planning Test Bench

When running the Dijkstra algorithm, a circle search is initiated, It can be seen that the maze navigator covers all possible areas of the maze. The color intensity of the each pixel creates circles, depending on the cost of that point to the starting point. The colors of the circles repeat given the limitations of the available colors; therefore, this does not mean that such pixels have the same cost.

Conversely, when we applied the Trémaux algorithm on the original maze, some loops are obtained in the trajectories. Thus, we adjusted the map that means we left each row and wall with the size of one pixel, which resulted in a bigger difference. Notice how the route of this maze seems more natural than the previous one. Most importantly, the robot does not have to walk everywhere in the maze, because the walls and paths are better defined. The intensity of each pixel represents how many times the robot has visited that node of the maze.

### 4.4 Communication Test Bench

Libraries that only work with *Python* 3.6 or higher were used in the camera calibration, mapping, and path planning modules, so the programming codes could not be executed locally, as the NAO is only programmable with some sub-version of *Python* 2.7. This would cause a delay in the development, because modules would have to be reprogrammed with some sub-version of *Python* 2.7, and even some libraries would not work due to incompatibility, for example, OpenCV. The solution to this paradigm was to run two *Python* scripts, one compiling with *Python* 2.7.17 and the other with *Python* 3.7.7. We opted to work using a master-slave architecture, where the computer is the master and the NAO robot (called Curie) is the slave.

Curie receives a script in *Python* 2.7.17 attending two tasks: 1) acquisition and storage of an image from the robot’s upper camera, and 2) its control i.e. moving or lifting the arms. In parallel, a *Python* 3.7.7 script was executed in another terminal on the same computer, which retrieved the stored images, processing them with one of the computational vision algorithms i.e. Canny. This approach solved the *Python* library compatibility issue.

### 4.5 Integration

**FIGURE 5:**
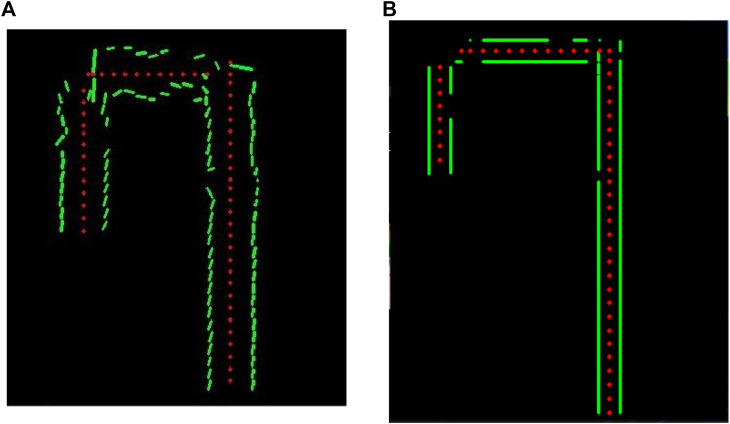
Sketch adjustment. **(A)** Map before adjustment. **(B)** Adjusted map.

Once all previous algorithms were selected and optimized, the integration of the four modules into a single system took place. Prior to this, it was decided to merge the mapping and path planning modules and validate them in the simulation environment. To do so, we had to consider as priority to draw the map lines completely straight (as in Figure 5B, in contrast to Figure 5A); always respecting the calculated distances discussed above. It was found that, by implementing this small change, the resulting algorithm was able to make more accurate decisions.

**FIGURE 6:**
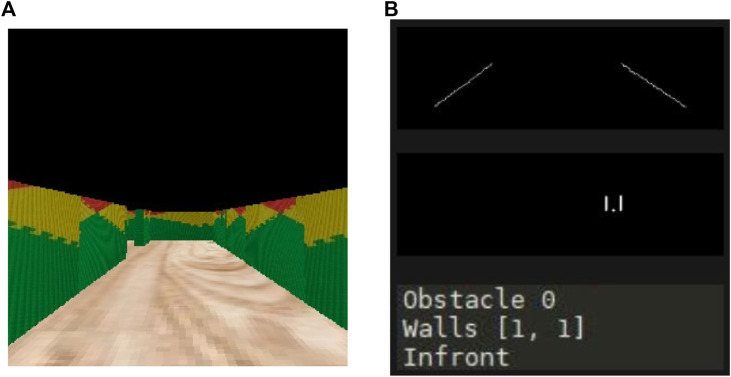
Mapping and path planning integration. First demo. **(A)** What the robot sees. **(B)** Algorithms output.

Figure 6 shows an example of these modules working together. It can be observed that, when using an image of a clear hallway as the one depicted in Figure 6A, the robot detects no obstacle and moves forward. Figure 6B illustrates the sketch of the stretch seen by the robot and the output obtained on the console, confirming that the system is working correctly.

**FIGURE 7:**
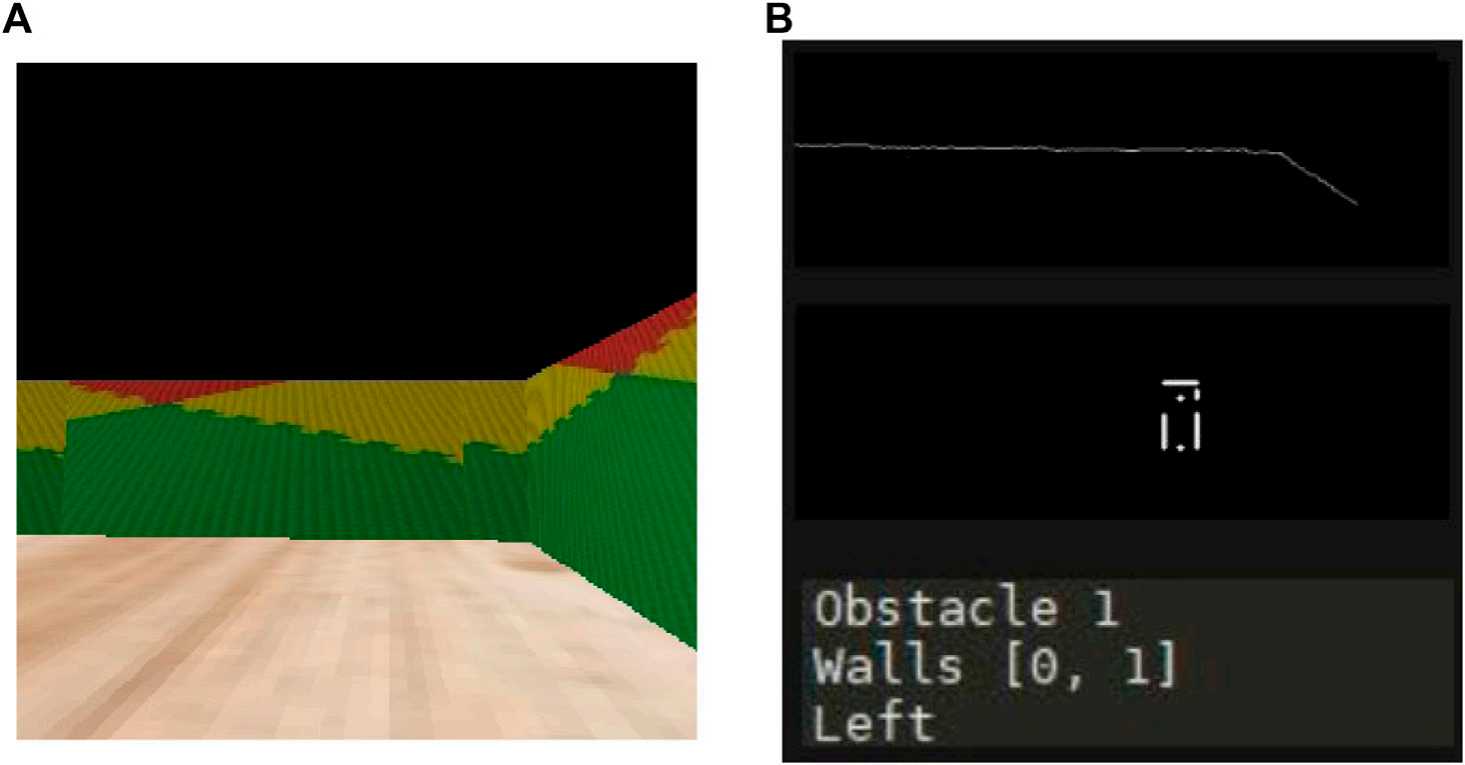
Mapping and path planning integration. Second demo. **(A)** What the robot sees. **(B)** Algorithms output.

Figure 7 presents another scenario where it can be seen that the robot is recognizing a corner as an obstacle, while at same time identifying that there is no wall to the left. As a result, the robot decides that the next action should be to turn left.

Once the integration of the path planning and mapping modules was achieved, the camera calibration was embedded into the NAO robot. This was evaluated with 24 photographs captured by Curie, where the center of the image is: (160*px*, 120*px*).

In general, it was observed that the photograph had deformations, both on the ROI (i.e. the chessboard) and the corners. The effects of these image deformations in the performance of the system will be detailed in Section 5.

A third integration took place during this phase, again with the path planning and mapping modules. This time, an adjustment had to be made to these modules to change the path from where it was retrieving the images. This means that images from the simulation were no longer in use, in favor of those taken by Curie. As all the modules were already integrated, a small labyrinth hall was built with foamy blocks and a yoga mat was placed as a floor, so that there was a greater contrast between the walls and the floor.

## 5 Analysis of Results and Discussion

### 5.1 Camera Calibration Analysis

As mentioned before, the calibration module was evaluated with 24 photographs captured by Curie, where the center of the image is: (160*px*, 120*px*) and the results obtained are shown in Table 2. It was observed that with 21 photographs, the lowest percentage of error was obtained when the center of the image was located with errors of 3.67*%* for the *x* axis and 5.6*%* for the *y* axis. As the number of photographs increased, so did the error, as evidenced by the results of using 24 photographs, where the errors obtained were 12.38 and 22.91*%* for the x and y coordinates respectively. The photographs presented considerable distortions, which affected the performance of the mapping and path planning modules considerably, given that the edges of the hall could not be correctly detected. This led to a higher contrast with the wall, even when using the yoga mat as the floor surface. We experimented removing the calibration module so that the mapping and path planning modules would directly process the photograph captured by Curie. Interestingly, this benefited the performance of the entire system, obtaining more acceptable performance. We suspect that this is due to the fact that the NAO robot already contains a calibration module embedded, and thus, this step is not required in this architecture. Nonetheless, it is important to realize that in other applications such as autonomous vehicles Cortés Gallardo Medina et al. (2021), this module must be considered.

**TABLE 2:** Comparative table between the centers of the real image retrieved from Curie and those of the matrix (best values highlighted in green).

Images	X	% Error X	Y	% Error Y
3	75.725	0.52671875	5.43	0.95475
6	92.115	0.42428125	14.55	0.87875
9	102.7	0.358125	24.59	0.795083333
12	117.375	0.26640625	39.815	0.668208333
15	128.21	0.1986875	62.8	0.476666667
18	148.915	0.06928125	82.9	0.309166667
21	154.125	0.03671875	113.18	0.056833333
24	179.81	0.1238125	147.5	0.229166667

### 5.2 Mapping Analysis

As mentioned in previous sections, three fundamental solutions had to be implemented in order to improve mapping. First, we had to stop mapping while orientation changes occurred to prevent the disruption of the sketch. Second, wall detection flags were required with two main purposes: 1) to avoid the computing of distances when a wall was not being detected, and; 2) to re enable the sketching after a turn took place. Finally, also in orientation changes, the center had to be adjusted to avoid overlapping the corner walls.

### 5.3 Path Planning Analysis

**FIGURE 8:**
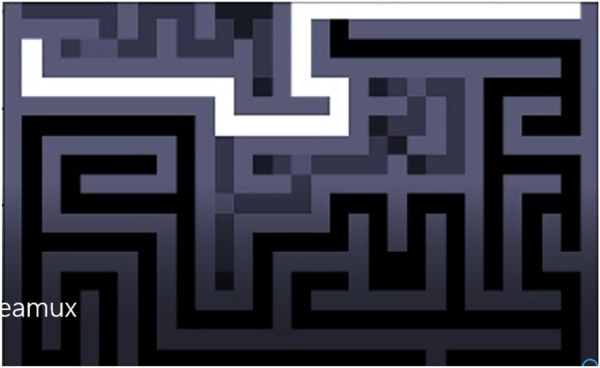
Trémaux algorithm: Path to the endpoint.

We discarded the Dijkstra algorithm because even if it is able to find the best path, there is still a need to traverse the whole maze, which is not a scalable solution, despite being an algorithm that does not need to know the endpoint and always finds an option for each position. Furthermore, It would be counterproductive to implement this solution in a robot, because it would force it to return to the beginning every time that the optimal distance has to be calculated. Therefore, the Trémaux algorithm, which demonstrated to be the fastest and most efficient method, was embedded into the model. Figure 8 shows that, even when the robot get into a dead end (as some situations depicted with gray pixels), it is also able to keep looking for the optimal path to the exit (white line).

### 5.4 Communication Analysis

In this module, we encountered no problems regarding loss of connection between Curie and the computer, or high latency in transmitting the flag generated by the mapping and path planning modules to Curie. The possible values of the flag based on the analysis carried out by the path planning and mapping modules (1–4), and the latency in transmitting this value to Curie was on average 333.12 *ms*. The path planning and mapping modules take approximately 10 s to perform all the processing of the photograph captured by Curie to throw the previously mentioned flag. Efforts were put to optimize the programming codes for both modules, unfortunately the time could not reduced.

### 5.5 Evaluation of the Module Integration

**FIGURE 9:**
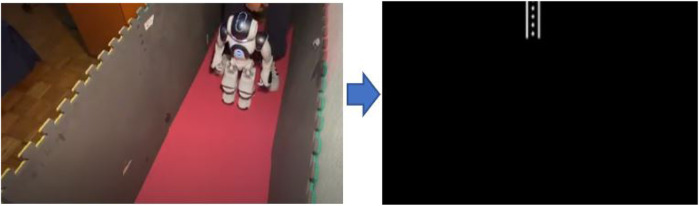
Test: straight path.

**FIGURE 10:**
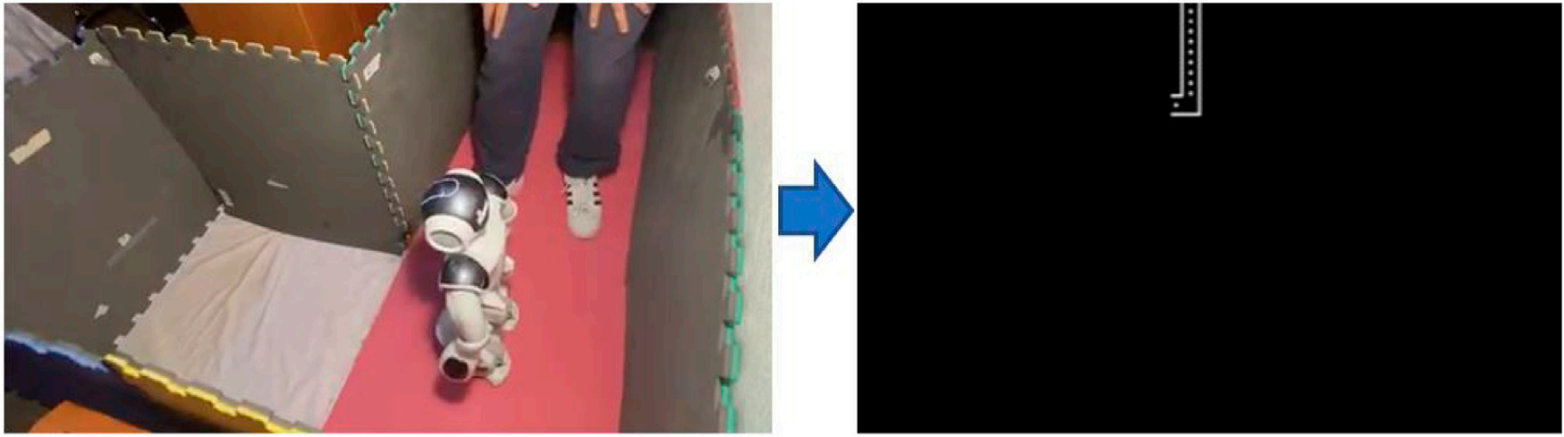
Test: left turn.

**FIGURE 11:**
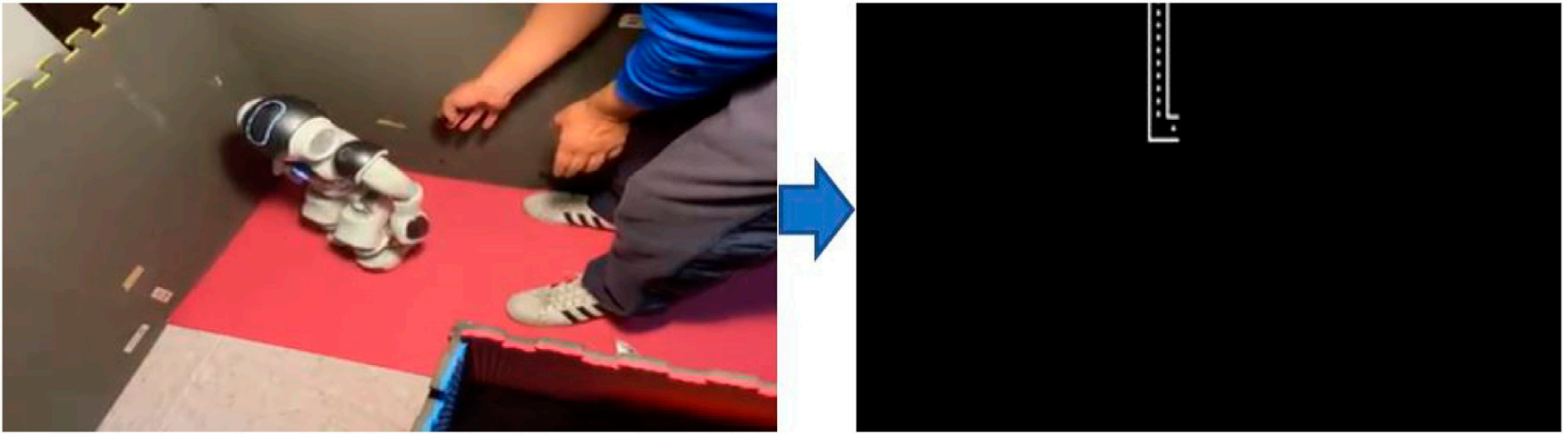
Test: right turn.

We ran the three following tests for our integrated system: 1) with a straight path as shown in Figure 9; 2) with a turn to the left as in Figure 10 and; 3) with a turn to the right as in Figure 11. All of these images show the robot traversing the path and how the maze is being sketch. In all three cases, the robot detected the walls and corners of the maze, which allowed it to follow the correct instruction. Also with our experimental results, we calculated that the time response between each robot action was about 10 s. Notice that the figures show that for these experiments, we used daylight lighting and we set up a maze using high-contrast surfaces and colors. Namely, the walls of the maze were gray and the floor is composed of red yoga mats. Moreover, there is no background noise or occlusions.

## 6 Conclusion and Future Work

In this paper, we presented our latest findings towards implementing a NAO robot maze navigation solution based on different algorithms, which range from computer vision, camera calibration, path planning, mapping, and communication protocols. With this, we aim at deploying a robot that is not only capable of solving a maze, but to share that knowledge with a second robot which will be also able to solve the maze considering its own mechanical characteristics. Several algorithms were tested and validated for each stage, showing the viability of this project.

The calibration module needs a second check, because at the moment, it is actually affecting the performance of the path planning and mapping instead of improve it. Nevertheless, base on the results of the test, we can infer that the NAO cameras are already sufficiently calibrated for the purpose of this task. Conversely, path planning, mapping and communication work really well together, but could be enhance by improving the response time of the robot to make it more continuous. Finally, we realized that uploading and retrieving information from Ubidots was what was causing the biggest delay on the performance. Once fixed, the NAO robot was capable of follow instructions based on its environment.

In future work, other path planning algorithms will be tested to further improve the time to solve the maze. We also need to do more tests with different maze structures and surfaces to see how the performance can be enhanced. Finally, at the communication stage, we will implement the aspect of sharing the generated knowledge with another robot, to take advantage of first robot’s experience. Moreover, we want to include the use of more features of the NAO robots which could improve the performance, in a similar way as presented in other work such as Almetwally and Mallem (2013). In this paper, author proposed the use of kinetic depth cameras and imitation learning. These algorithms require more work and computational resources, nonetheless, by learning from human motion and activity, it is highly likely that the NAO robots are able to better navigate throughout the maze.

Furthermore, we also want to explore the application of these technologies for educational purposes. As the COVID-19 global pandemic has shown us, social distancing and remote learning will be the reality of many people of us for the foreseeable future. Therefore, having robot-assisted systems which can show certain activities remotely to train personnel (avoiding human contact) is of utmost important in settings such as the industry or care homes for the vulnerable.

## Data Availability

The original contributions presented in the study are included in the article/Supplementary Material, further inquiries can be directed to the corresponding author.

## Footnotes

1
https://www.softbankrobotics.com/emea/en/nao

2
http://doc.aldebaran.com/2-1/family/index.html

3
https://opencv.org/

4
https://www.coppeliarobotics.com/helpFiles/index.html

5
https://stackoverflow.com/questions/45531074/how-to-merge-lines-after-houghlinesp

6
https://github.com/ndrplz/self-driving-car

7
https://www.iana.org/form/ports-services

8
https://scikit-image.org/docs/dev/auto_examples/edges/plot_canny.html
